# Trends in Syphilis Incidence and Its Association With the Number of Sex Industry-Related Businesses in Japan: An Ecological Study Using Joinpoint Analysis

**DOI:** 10.7759/cureus.83294

**Published:** 2025-05-01

**Authors:** Yoshiro Mori, Nobuyuki Miyatake, Yuka Mori, Kiyotaka Tanimoto, Hisayoshi Morioka

**Affiliations:** 1 Department of Public Health, Graduate School of Biomedical Sciences, Tokushima University, Tokushima, JPN; 2 Department of Hygiene, Faculty of Medicine, Kagawa University, Miki, JPN; 3 Graduate School of Biomedical Sciences, Tokushima University, Tokushima, JPN; 4 Department of Infectious Diseases, Sakaide City Hospital, Sakaide, JPN

**Keywords:** hiv/aids, joinpoint analysis, public health policy, sexually transmitted infections, syphilis trends

## Abstract

Background: In recent years, a notable increase in syphilis cases has been observed in Japan, aligning with global trends. We aimed to elucidate the trends in sexually transmitted infections (STIs) incidence (specifically syphilis) in Japan and examine its relationship with the number of sex industry-related businesses in prefectures.

Methods: Data on STIs and the number of sex industry-related businesses from 2000 to 2022 were obtained from the National Institute of Infectious Diseases (NIID) and the official website of the Japan National Crime Prevention Association, respectively. Joinpoint analysis and regression analysis were used to identify inflection points in the trend of syphilis and describe the association between STI incidence and the overall number (and the number of different categories) of sex industry-related businesses (i.e., store-based soaplands, store-based fashion health, nude studio, love hotels, adult shops, and meeting cafes, and non-store-based including dispatch-type fashion health and adult video mail orders). The population data was obtained from the 2022 population estimates provided by the Statistics Bureau of the Ministry of Internal Affairs and Communications.

Results: In this study, a significant increase in the number of syphilis cases has been observed in Japan in recent years, with joinpoint analysis identifying a significant inflection point in 2020 (annual percent change: 43.8). Multiple regression analysis adjusted for the prefectural population revealed a strong association between the incidence of STIs and number of sex industry-related businesses. Specifically, a strong association was observed between the number of Category I non-store-based sex-related business syphilis cases among men (standardized β=0.908, p<0.001, R^2^=0.938) and women (standardized β=1.017, p<0.001, R^2^=0.921), as well as AIDS cases for men (standardized β=0.991, p<0.001, R^2^=0.910).

Conclusions: The significant increase in syphilis cases in Japan among men and women may be related to an increase in non-store-based, dispatch-type fashion health sex-related businesses. These findings indicate that public health policies should consider these specific social and behavioral factors in future interventions.

## Introduction

Sexually transmitted infections (STIs) include conditions such as genital chlamydia, genital herpes, condyloma acuminatum, acquired immunodeficiency syndrome (AIDS), syphilis, and gonococcal infections, which are potentially transmitted through sexual contact. In Japan, syphilis and AIDS are monitored comprehensively, while genital chlamydia, genital herpes, condyloma acuminatum, and gonorrhea are monitored through sentinel surveillance. Syphilis, one of the representative STIs caused by the bacterium *Treponema pallidum*, has seen an increase in cases reported globally in recent years [1]. This trend is also observed in Japan, where the rising number of syphilis cases is a growing concern [2].

According to reports from the National Institute of Infectious Diseases (NIID), the number of syphilis cases in Japan has steadily increased over the past decade, surpassing 10,000 cases in 2022. This significant rise suggests potential issues in public health management and sexual education [3]. Globally, factors contributing to this increase have included the development of social networking services and dating websites via smartphones [4], as well as outbreaks among men who indulge in sex with men [5]. Therefore, understanding the factors contributing to the rise in syphilis cases is crucial for future syphilis control measures. Although providing sexually oriented goods and services, the sex industry is the subject of numerous intense social and ethical debates, and from a public health perspective, it is particularly important in relation to STIs [6,7]. The risk of STIs is heightened owing to routine sexual contact with multiple partners. Although consistent condom use and regular testing are essential for the prevention of STIs, comprehensive prevention measures across the industry remain a challenge [8].

Previous studies have evaluated changes in STI incidence in various populations in Japan using a descriptive analysis [9,10]. Our present study used joinpoint regression analysis, which assesses changes in time-series data to identify the timing of these changes. We elucidated the trends in STI incidence (specifically syphilis) in Japan by using joinpoint analysis and examined its relationship with the number of sex industry-related businesses in 47 prefectures to inform STI control efforts in Japan through an ecological study.

## Materials and methods

Study setting

This study was conducted across all 47 prefectures of Japan. The primary period of analysis was the year 2022. Additionally, historical trends of syphilis cases were examined from 2000 to 2022.

Study design

We conducted a cross-sectional ecological study to investigate the association between the number of sex-related businesses and the number of STI cases at the prefectural level in 2022. Furthermore, a time trend analysis of syphilis cases was performed using joinpoint regression analysis over the period from 2000 to 2022.

Study unit

The unit of analysis was each prefecture. Aggregated prefecture-level data were used for STI case numbers, sex-related business counts, and population statistics.

Data collection

STI Data Sources and Surveillance Methods

In this study, we used the latest STI data (from 2000 to 2022) provided by the NIID [11]. The STIs included, based on the representative diseases classified by the NIID, were syphilis, AIDS, genital chlamydia, genital herpes, condyloma acuminatum, and gonococcal infection. The NIID database provided data on the total number of cases and cases stratified by sex, but did not include information on the age of individuals. “Total Count” refers to a method in which all cases of infection are reported by every medical institution and clinic, which applies to syphilis and AIDS [11]. “Sentinel Surveillance” refers to a method in which selected medical institutions and clinics report the occurrence of infections, and the overall infection status is estimated based on these reports, which applies to genital chlamydia, genital herpes, condyloma acuminatum, and gonococcal infection [12].

The selection of sentinel medical institutions is conducted according to the national guidelines, specifically the "Implementation Guidelines for the National Epidemiological Surveillance of Infectious Diseases."

In principle, sentinel surveillance is randomly selected from available medical institutions with cooperation from local medical associations, and chosen by considering the population and geographic distribution of healthcare facilities to ensure representative monitoring of the entire prefecture.

Thus, sentinel surveillance is designed to minimize selection bias and accurately reflect the epidemiological trends of infectious diseases across the country.

Sex-Related Business Types and Data Collection

The latest data (2022) on sex-related businesses was obtained from the official website of the National Social Environment Management Association, a subsidiary of the Japan National Crime Prevention Association [13]. Definitions of each business type were referenced from the definitions provided by the National Police Agency regarding sex-related businesses [14]. These definitions are detailed in Table 1.

**Table 1:** Definition of each business type

Sex-Related Business Types	Category		Definition
Store-based sex-related businesses	Category I	Soaplands	These businesses establish private rooms as part of a bathhouse and provide services involving physical contact with customers of the opposite sex.
	Category II	Store-based fashion health	These businesses establish private rooms and provide services involving physical contact with customers of the opposite sex according to their sexual interests (excluding businesses that fall under Category I).
	Category III	Nude studios, private video booths, peep shows, strip theaters, etc.	These businesses exclusively provide performances where individuals remove their clothing to arouse sexual interest or other performances that have a significantly negative impact on public morals or the sound development of youth, as specified by government ordinances.
	Category IV	Love hotels, motels, rental rooms	These businesses establish facilities specified by government ordinances, exclusively used for the accommodation (including rest) of customers accompanied by members of the opposite sex, and allow such facilities to be used for this purpose.
	Category V	Adult shops, sex toy stores, etc.	These businesses establish stores to sell or rent items specified by government ordinances, such as photographs, videotapes, and other products that arouse sexual interest.
	Category VI	Meeting cafes	These establishments are defined as businesses that set up stores where individuals who wish to satisfy temporary sexual curiosity with unfamiliar members of the opposite sex can engage in interactions, including conversations. The store facilitates this by either introducing the individuals after they have viewed each other’s appearance or images within the premises, or by providing opportunities for meetings in private rooms or similar facilities within the store.
Non-store-based sex-related businesses	Category I	Dispatch-type fashion health, etc.	These are businesses that provide services involving physical contact with customers of the opposite sex, according to their sexual interests, in locations such as residences or facilities used for accommodation. The services are conducted by dispatching personnel to the customer upon their request.
	Category II	Adult video and similar products, mail-order sales	These businesses involve selling or renting items specified by government ordinances, such as photographs, videotapes, and other products that arouse sexual curiosity, upon receiving customer orders via phone or other means. The items are delivered to the customer or arranged to be delivered.

In this study, we focused on business types that are considered to have a high likelihood of sexual intercourse and selected Category I of store-based sex-related businesses (soaplands), Category II (store-based fashion health), Category IV (love hotels, motels, rental rooms), and Category I of non-store-based sex-related businesses (dispatch-type fashion health, etc.). These business types were considered to have a higher likelihood of direct sexual contact and, therefore, a relatively higher risk of STIs compared to other business types (Categories III, V, VI of store-based businesses and Category II of non-store-based businesses). Additionally, Categories III, V, and VI of store-based sex-related businesses, as well as Category II of non-store-based sex-related businesses, were excluded from this analysis as they are primarily associated with visual sexual stimulation or product sales, which are not directly related to the risk of STIs.

Population Data

The population data for each prefecture in Japan was obtained from the 2022 population estimates provided by the Statistics Bureau of the Ministry of Internal Affairs and Communications [15].

Accessibility of Data

All datasets used in this study were sourced from publicly available databases in Japanese, accessible to anyone without restriction.

Data analysis

The annual number of reported cases of STIs was identified, and the number of infected cases and the number of sex industry-related businesses in each prefecture in 2022 are expressed as median and interquartile range (IQR). The trend in syphilis cases from 2000 to 2020 was analyzed using joinpoint analysis [16] to identify inflection points. Multiple linear regression was performed using the number of different types of sex-related businesses in 2022 (Category I store-based, Category I non-store-based, Category II, and Category IV) and the population of each prefecture as the independent variable, and the number of STIs in 2022 as the dependent variable. In each statistical analysis, a significance level set at p < 0.05 was set. Data analysis was performed using the Joinpoint Regression Program (provided by the National Cancer Institute, USA) and JMP statistical software (JMP Pro version 17, SAS Institute Inc., Cary, NC, USA).

Ethical approval

All data used in this study were obtained from the official websites. As this study utilized open data, individual informed consent was not required. Ethical approval was obtained from the Ethical Committee of Sakaide City Hospital in Sakaide, Japan (approval no.: 2024-002, date: April 30, 2024). The committee carefully reviewed and approved the study methods, confirming that the study adhered to ethical guidelines and ensured the protection of participants' rights and privacy.

## Results

**Figure 1:**
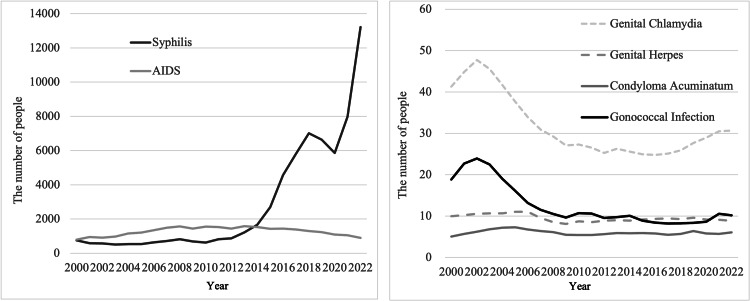
Sexually transmitted infections (STIs) over the past 22 years Syphilis and AIDS counts are from total national surveillance. Genital chlamydia, genital herpes, condyloma acuminatum, and gonococcal infection data are from sentinel surveillance systems.

**Figure 2:**
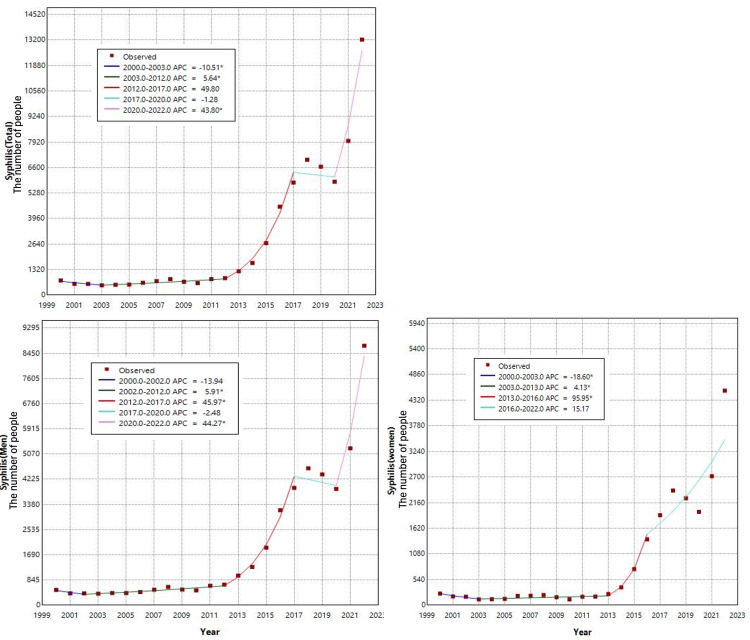
Joinpoint regression analysis of syphilis from 2000 to 2022 * indicates statistically significant (p < 0.05) by joinpoint regression analysis. APC: annual percent change

The 2022 data on STIs and the number of sex-related businesses across all 47 prefectures in Japan are summarized in Tables 2, 3. The trends in the number of STI cases from 2000 to 2022 were captured through descriptive statistics (Figure 1). Notably, a significant increase in syphilis cases was observed in recent years. Although genital chlamydia showed a slight upward trend, other infections remained relatively stable over the period. Due to the marked increase in syphilis cases, we performed a joinpoint analysis to identify inflection points (Figure 2). A significant inflection point was observed in 2020 for the overall population (annual percent change (APC): 43.8) and for men (APC: 44.27). For women, a significant inflection point was observed in 2013 (APC: 95.95).

**Table 2:** Median number of STIs per prefecture in Japan in 2022 IQR: interquartile range; AIDS: acquired immunodeficiency syndrome; STIs: sexually transmitted infections Number of prefectures: 47 Syphilis and AIDS counts are from total national surveillance. Genital chlamydia, genital herpes, condyloma acuminatum, and gonococcal infection data are from sentinel surveillance systems.

Infection Type	Category	Median	IQR
Syphilis	Total	120.0	(58.0-240.0)
	Men	75.0	(40.0-194.0)
	Women	35.0	(15.0-58.0)
AIDS	Total	6.0	(3.0-16.0)
	Men	6.0	(3.0-14.0)
	Women	0.0	(0.0-1.0)
Genital chlamydia	Total	25.5	(18.0-38.5)
	Men	12.9	(6.6-21.3)
	Women	12.0	(7.7-17.4)
Genital herpes	Total	8.3	(5.1-12.6)
	Men	2.3	(0.9-4.4)
	Women	5.0	(3.4-7.3)
Condyloma acuminatum	Total	4.4	(2.9-6.7)
	Men	2.9	(1.4-4.9)
	Women	1.5	(1.1-2.2)
Gonococcal infection	Total	8.5	(4.6-12.9)
	Men	6.2	(3.2-10.0)
	Women	1.8	(0.9-2.5)

**Table 3:** Median number of sex-related businesses per prefecture in Japan in 2022 IQR: interquartile range Number of prefectures: 47

	Category	Median	IQR
Non-store-based sex-related businesses	Category Ⅰ	304	(139-514)
Store-based sex-related businesses	Category Ⅰ	9	(2-40)
	Category Ⅱ	0	(0-12)
	Category Ⅳ	83	(54-145)

The results of the multiple regression analysis, which were adjusted for population, are shown in Table 4. In this analysis, the number of sex-related businesses and the sex-specific population of each prefecture were used as independent variables, whereas the number of patients with each STI served as the dependent variable. Syphilis emerged as a significant determinant associated with the number of Category I non-store-based sex-related businesses for men (standardized β=0.908, p<0.001, R^2^=0.938) and women (standardized β=1.017, p<0.001, R^2^=0.921). Additionally, the number of Category II store-based businesses was a significant determinant for syphilis in women (standardized β=-0.190, p=0.046, R^2^=0.718). No other business categories showed significant associations when adjusted for population.

**Table 4:** Associations between the number of reported STIs and the number of sex industry-related businesses: results from multiple regression analysis Bold values indicate p<0.05 by multiple regression analysis. STIs: sexually transmitted infections

Infection Type	Category Type	Men			Women		
		R^2^	Standardized β	p-value	R^2^	Standardized β	p-value
Syphilis	Category Ⅰ Non-store-based sex-related businesses	0.938	0.908	<0.001	0.921	1.017	<0.001
	Category Ⅰ Store-based sex-related businesses	0.763	0.191	0.070	0.697	0.109	0.359
	Category Ⅱ Store-based sex-related businesses	0.762	-0.153	0.081	0.718	-0.190	0.046
	Category Ⅳ Store-based sex-related businesses	0.751	0.102	0.323	0.698	0.110	0.335
AIDS	Category Ⅰ Non-store-based sex-related businesses	0.910	0.991	<0.001	0.557	-0.124	0.562
	Category Ⅰ Store-based sex-related businesses	0.732	0.319	0.006	0.558	0.095	0.507
	Category Ⅱ Store-based sex-related businesses	0.700	-0.165	0.091	0.561	0.096	0.409
	Category Ⅳ Store-based sex-related businesses	0.684	0.084	0.466	0.557	-0.080	0.562
Genital Chlamydia	Category Ⅰ Non-store-based sex-related businesses	0.037	0.187	0.545	0.115	0.380	0.214
	Category Ⅰ Store-based sex-related businesses	0.041	0.154	0.463	0.091	0.126	0.538
	Category Ⅱ Store-based sex-related businesses	0.029	0.002	0.990	0.109	0.187	0.264
	Category Ⅳ Store-based sex-related businesses	0.060	0.239	0.233	0.351	0.704	<0.001
Genital Herpes	Category Ⅰ Non-store-based sex-related businesses	0.040	0.270	0.381	0.019	0.279	0.383
	Category Ⅰ Store-based sex-related businesses	0.031	0.125	0.554	0.006	-0.093	0.665
	Category Ⅱ Store-based sex-related businesses	0.029	0.086	0.619	0.016	0.141	0.420
	Category Ⅳ Store-based sex-related businesses	0.046	0.204	0.311	0.102	0.432	0.031
Condyloma Acuminatum	Category Ⅰ Non-store-based sex-related businesses	0.286	0.683	0.013	0.492	0.781	0.001
	Category Ⅰ Store-based sex-related businesses	0.190	0.160	0.405	0.376	0.197	0.248
	Category Ⅱ Store-based sex-related businesses	0.179	-0.059	0.710	0.409	-0.264	0.055
	Category Ⅳ Store-based sex-related businesses	0.186	0.127	0.494	0.372	0.170	0.302
Gonococcal Infection	Category Ⅰ Non-store-based sex-related businesses	0.132	0.390	0.186	0.383	0.929	0.001
	Category Ⅰ Store-based sex-related businesses	0.108	0.152	0.453	0.197	0.103	0.595
	Category Ⅱ Store-based sex-related businesses	0.100	0.068	0.683	0.208	0.146	0.353
	Category Ⅳ Store-based sex-related businesses	0.166	0.357	0.062	0.332	0.509	0.004

AIDS cases demonstrated a significant correlation with the number of Category I non-store-based sex-related businesses in men (standardized β=0.991, p<0.001, R^2^=0.910). Furthermore, the number of Category I store-based businesses was identified as a significant determinant for AIDS exclusively in men (standardized β=0.319, p=0.006, R^2^=0.732). Genital chlamydia was significantly associated with the number of Category IV store-based sex-related businesses, but only in women (standardized β=0.704, p<0.001, R^2^=0.351). Genital herpes was significantly associated with the number of Category IV store-based sex-related businesses in women (standardized β=0.432, p=0.031, R^2^=0.102). Condyloma acuminatum was significantly associated with the number of Category I non-store-based sex-related businesses for men (standardized β=0.683 (p=0.013, R^2^=0.286) and women (standardized β=0.781 (p=0.001, R^2^=0.492). Gonococcal infection was significantly associated with the number of Category I non-store-based sex-related businesses in women (standardized β=0.929, p=0.001, R^2^=0.383). Additionally, the number of Category IV store-based sex-related businesses was a significant determinant for gonococcal infection in women (standardized β=0.509, p=0.004, R^2^=0.332).

## Discussion

In this study, we evaluated the epidemiological trends of syphilis and other STIs in Japan, exploring their relationship with the prevalence of sex-related businesses through an ecological analysis. The findings revealed a significant inflection point in syphilis cases in 2020 among both the overall population and men, identified through joinpoint analysis. Notably, a strong association was observed between syphilis incidence and the number of Category I non-store-based sex-related businesses in both men and women.

The utility of joinpoint analysis in epidemiological studies is well-documented, as evidenced by Guo et al., who applied this method to analyze trends in cervical cancer mortality in China [17], and Wu et al., who used it to assess global trends in cirrhosis incidence [18]. In the domain of STIs, Wang et al. employed joinpoint analysis to examine trends in STIs and blood-borne infections in China [19], whereas Molnar et al. utilized it to assess syphilis and gonorrhea trends among adolescents in Romania [20]. These studies underscore the robustness of joinpoint analysis in detecting significant shifts in epidemiological trends, thereby facilitating the investigation of underlying factors.

Although other STIs, such as AIDS, did not exhibit a significant upward trend, syphilis demonstrated a notable increase in recent years, with a significant inflection point detected in 2020 for both the overall population and men. This rise may be partly attributable to behavioral changes induced by the COVID-19 pandemic, which likely led to a decline in the utilization of sex-related services. Concurrently, there has been a reported decrease in store-based sex-related businesses and a corresponding increase in non-store-based sex-related businesses [21]. By contrast, a significant inflection point in syphilis cases among women was observed in 2013, potentially linked to the rapid proliferation of dating apps in Japan around 2012 [3]. Research by Allen et al. has elucidated the relationship between mobile and internet use and STI incidence, whereas Chan et al. reported associations between dating app usage and STI rates [22,23]. In Japan, Suzuki et al. further identified a correlation between the rise in syphilis cases and the use of dating apps [3], though the specific mechanisms remain unclear.

Our study reveals a strong association between syphilis incidence and the number of Category I non-store-based sex-related businesses in both men and women. While store-based sex-related businesses are subject to direct audits and inspections, allowing for better monitoring of hygiene conditions, non-store-based businesses pose a greater challenge for regulatory oversight, as inspections are typically confined to administrative offices rather than service locations. Moreover, the differences in the infectivity of each STI cannot be ignored. It has been reported that the probability of HIV transmission per sexual act is generally between 0.1% and 1% [24]. However, the probability of syphilis transmission per sexual act has been reported as 30% by Schroeter et al. [25]. Additionally, while condoms are considered to reduce the risk of HIV (AIDS) transmission by approximately 80% [26], syphilis, which can enter the bloodstream through microabrasions in the skin or mucous membranes, has a much lower prevention efficacy with condoms, reported at 39% [27]. These factors, combined with varying levels of regulatory oversight, likely contribute to the differential associations observed between STIs and the various categories of sex-related businesses.

These factors, including the level of surveillance by regulatory authorities based on law and the differences in the infectivity of the diseases themselves, likely contribute to the observed differences in the correlation between STIs and the number of sex-related businesses in various categories. The findings of this study provide valuable public health insights that could be used in the control of syphilis and other STIs in Japan. Strengthening the regulation and oversight of non-store-based sex-related businesses, particularly those offering direct services, could be pivotal in reducing infection risks. Additionally, it has been reported that 4% of women and 48.3% of men have used commercial sex workers (CSW) in Japan, with 5.3% of women and 59.6% of men in the 20-29 age group and the 40-49 age group being the most frequent users of CSW [28], expanding testing and early treatment, supporting those infected, reducing social stigma, and enhancing sexual education, especially among young individuals, are critical measures needed to curb the spread of these infections [29,30].

Limitations

This study has certain limitations. First, as an ecological study, it does not assess individual-level data, limiting the ability to generalize findings to individual cases. Second, the number of sex-related businesses may not fully reflect actual operational activities, and the potential influence of unlicensed operations must be considered. Third, evolving changes in sexual behavior, STI testing rates, and health-seeking behavior - factors not accounted for in our analysis - may have influenced our study findings. Fourth, this study has not addressed STIs among transgender individuals and homosexual populations. Despite these limitations, we believe that our findings offer significant public health insights into the control of syphilis and other STIs in Japan. Comprehensive strategies, particularly targeting non-store-based sex-related businesses, could be instrumental in controlling the spread of syphilis.

## Conclusions

The findings of this study underscore the significant increase in syphilis cases in Japan, particularly highlighting a possible association with specific social and behavioral factors, notably the number of Category I non-store-based sex-related businesses. These results indicate a pressing need for public health policies that incorporate these factors into future intervention strategies. Future research could conduct population-based surveys or surveys on persons working in the sex industry to incorporate various confounding factors and validate these findings.
